# Involvement of the contact pathway in COVID-19 coagulopathy

**DOI:** 10.1007/s11739-025-04191-z

**Published:** 2025-11-08

**Authors:** Marco Capecchi, Cristina Novembrino, Maria Abbattista, Massimo Boscolo-Anzoletti, Eleonora Galbiati, Samantha Griffini, Elena Grovetti, Luca Valenti, Francesco Blasi, Giacomo Grasselli, Roberta Gualtierotti, Massimo Cugno, Flora Peyvandi

**Affiliations:** 1 Angelo Bianchi Bonomi Hemophilia and Thrombosis Center, Fondazione IRCCS Ca’ Granda Ospedale Maggiore Policlinico, Milan, Italy; 2 Division of Hematology, Clinica Moncucco, via Soldino 5, 6900 Lugano, Switzerland; 3 Department of Transfusion Medicine, Biological Resource Center, Fondazione IRCCS Ca’ Granda Ospedale Maggiore Policlinico, Milan, Italy; 4 Respiratory Unit and Cystic Fibrosis Adult Center, Fondazione IRCCS Ca’ Granda Ospedale Maggiore Policlinico, Milan, Italy; 5 Dipartimento di Anestesia, Rianimazione e Emergenza-Urgenza, Fondazione IRCCS Ca’ Granda Ospedale Maggiore Policlinico, Milan, Italy; 6 Department of Pathophysiology and Transplantation, Università degli Studi di Milano, Milan, Italy

**Keywords:** Contact pathway, COVID-19 coagulopathy, Extrinsic pathway, Thromboinflammation, Thrombosis

## Abstract

A novel acquired coagulopathy characterized by severe procoagulant imbalance is common and associated with the clinical severity in COVID-19 patients. To elucidate the underlying mechanisms of coagulation activation in COVID-19 patients. Symptomatic COVID-19 patients were consecutively enrolled and stratified into 3 groups based on the intensity of care. Markers of intrinsic (FXIa, FXIIa) and extrinsic (FVIIa) pathway activation and of fibrinolysis (plasminogen and relative activator and inhibitors), d-dimer, fibrin monomer (FM), fibrin degradation products (FDP), and C1 inhibitor were tested. A total of 111 patients were enrolled, 26 in the low, 42 in the intermediate, and 43 in the high intensity of care group. Median d-dimer, FDP, and FM plasma levels were higher in COVID-19 patients than normal ranges, with a gradient of increase across the three intensity care units; the fibrinolytic pathway parameters were in the normal range. The median plasma levels of FVIIa were lower in COVID-19 patients (27.5 mU/mL) than the reference range while the median plasma levels of FXIIa and FXIa were higher (11.2 and 11.3 mU/mL), with a gradient of increase across the three intensity care units for FXIIa. C1 inhibitor plasma levels were above the normal range in all the 3 COVID-19 patient groups. 32 patients (29%) developed a venous thrombosis. Our study suggested a prevalent activation of the contact pathway over the extrinsic pathway of the coagulation cascade in COVID-19 patients, which is proportional to the clinical severity of the infection, opening the possibility for targeted anticoagulant therapies.

**Supplementary Information:**

The online version contains supplementary material available at https://doi.org/10.1007/s11739-025-04191-z.

## Introduction

Coronavirus disease 2019 (COVID-19), caused by severe acute respiratory syndrome coronavirus 2 (SARS-CoV-2), is characterized by an extremely heterogeneous clinical presentation, ranging from asymptomatic to critically ill patients. Around 5% of patients with an acute respiratory distress syndrome (ARDS) can occur and may progress to respiratory failure with a multi-organ damage and death [1] (fatality rate from 2.3 to 49%). The most common risk factor for mortality (around 10% of fatal cases) was represented by a pre-existing cardiovascular disease [2, 3]. During the first wave of the pandemic, thrombotic complications, mainly venous, but also arterial and microvascular, have been reported in hospitalized patients with an incidence varying from 26 to 75% [4]. The highest figures were reported in patients with more severe disease, modulated by the contribution of variability at some genetic loci associated with lung function and mucosal barrier or autoimmune and inflammatory diseases [5, 6]. Pulmonary embolism accounted for up to 80% of the thrombotic complications in a series of ICU patients, being disproportionately high compared to the number of deep venous thrombosis, therefore suggesting primary in situ thrombosis as the underlying mechanism, rather than embolism [7]. Thrombotic complications have been reported to be the second cause of mortality after respiratory failure. In July 2020, the first guideline based on randomized controlled trials (RCTs) on anticoagulant prophylaxis in COVID19 patients has been released by the International Society on Thrombosis and Haemostasis. According to these guidelines, prophylactic dose heparin is recommended for both critically ill and non-critically ill hospitalized patients while therapeutic dose heparin is recommended only for selected non-critically ill patients with elevated d-dimer levels and increased oxygen requirements without an increased bleeding risk. Intermediate dose heparin is not recommended in any subset of patients, in contrast to previous suggestions [8]. Of note, most of the RCTs considered for developing these guidelines enrolled patients during the first waves of the pandemic with the initial variants of SARS-CoV-2 and before anti-SARS-CoV-2 vaccination became widely available. Therefore, these recommendations should be used with caution in other settings. A concomitant high risk of bleeding has also been reported in these patients while on anticoagulant treatment [9], making decisions on antithrombotic prophylaxis challenging.

The role of d-dimer levels as a prognostic marker has been extensively investigated. It has demonstrated the association between elevated d-dimer levels and an increased risk of mortality [10] and d-dimer at admission has been shown to be a dose-dependent predictor of venous thromboembolism (VTE) occurrence during admission or within 14 days after hospital discharge.

The increased risk of thrombosis in patients with COVID-19 is associated with a pattern of coagulopathy that shares some characteristics with other coagulopathies, such as disseminated intravascular coagulation (DIC), or sepsis-induced coagulopathy (SIC), but has unique features. These include increased d-dimer levels without reduced fibrinogen levels and markedly increased factor VIII (FVIII) and von Willebrand factor (VWF) plasma levels [11].

Several mechanisms by which SARS-CoV-2 activates the coagulation cascade have been identified [12], most of which are secondary to the inflammatory response induced by the virus. Both the virus itself and the induced systemic inflammation are responsible for the endothelial perturbation with a consequent increased expression of procoagulant factors, such as tissue factor and VWF, and reduced expression of anticoagulant factors, such as ADAMTS13 and thrombomodulin [13]. Together with an activation of the coagulation cascade, a reduced activity of the fibrinolytic system has been reported [14]. The predominant involvement of endothelial cells is demonstrated by the fact that circulating parameters of endothelial derangement, such as circulating endothelial cells, circulating endothelial progenitor cells, endothelial extracellular vesicles, and soluble forms of endothelial adhesive proteins, are strongly altered in COVID-19 patients [15].

COVID-19 is characterized by a unique coagulopathic profile. Indeed, the hemostatic abnormalities seen in COVID-19 are distinct from those observed in both bacterial sepsis and other viral infections. COVID-19 displays a unique interplay between inflammation and hypercoagulability, with thrombosis being a major clinical feature. This contrasts with the classic DIC of bacterial sepsis and the hemorrhagic tendencies of viral hemorrhagic fevers [16, 17].

The contact/intrinsic pathway of the coagulation cascade may play a crucial role in coagulation activation in COVID-19 due to its deep interconnection with pathways involved in the inflammation response, such as the complement and the kallikrein–bradykinin systems, considering the extensive inflammatory response that characterizes SARS-CoV-2 infection [18]. By inducing platelets activation and their consequent interaction with cells of the innate immune system, such as neutrophils, SARS-CoV-2 give rise to neutrophil extracellular traps (NETs) release [19]. Indeed, markers of NETS formation, such as myeloperoxidase and citrullinated histone H3, have been found to be increased in COVID-19 patients [20]. Moreover, impairment of the endogenous deoxyribonucleases (DNases) activity, which normally clear NETs from blood circulation and tissues, results in defective NETs clearance and their accumulation in tissues. FXIIa have been found to co-localize with NETs in autopsies studies on COVID-19 patients, suggesting that NETs may provide a scaffold for FXII activation [21]. Therefore, defective NETs clearance in COVID-19 may contribute to sustained FXII activation.

Given this background, we hypothesized that the coagulopathy of patients with severe COVID-19 is caused by a predominant activation of the contact pathway of the coagulation cascade over the extrinsic pathway. The aim of this study was therefore to examine the differential activation of the extrinsic and the intrinsic pathways of the coagulation cascade in patients hospitalized due to severe SARS-CoV-2 infection.

## Methods

### Study design and population

This is a cross-sectional study on consecutive COVID-19 patients admitted to our hospital between February and April 2020. This study was conducted according to the 2013 revision of the Declaration of Helsinki and the code of Good Clinical Practice and approved by the local ethic committee of Milano—Area 2 (No. 360_2020). All patients provided the written informed consent before any study procedure. Depending on the severity of the disease and consequent degree of oxygen need, they were admitted to low-intensity care when hypoxia was handled by ventilation support with high-flow nasal cannulas; to intermediate sub-intensive care when hypoxia prompted the use of continuous positive airway pressure; or to high-intensity care when hypoxia warranted intubation and mechanical ventilation in ICU. Each patient was included in the study only once, so that in case of switch from one severity class to another, the same patient was not considered again in the study to avoid not independent observations.

All patients started antithrombotic prophylaxis with low-dose low molecular weight heparin (LMWH) at admission and dosages were then adjusted by attending physicians after their transfer to the hospital wards. In low-intensity care wards, patients were treated with enoxaparin 70 UI/Kg once daily; in an intermediate intensity with 70 UI/kg twice daily; in a high intensity with 100 UI/kg once daily. Blood samples were collected at least 72 h after the start of LMWH prophylaxis and before the administration of the daily dose. Patients that showed a congenital deficit of FVII, FXI, or FXII were excluded from subsequent analysis. Patients were not systematically screened for venous thrombosis, but the diagnosis was symptom-driven throughout the inward permanence.

### Laboratory methods

Venous blood was collected by venipuncture in vacuum tubes containing 1/10 volumes of tri-sodium citrate 0.109 M. Specimens were centrifuged for 20 min at 3000×*g* within 2 h and the supernatant was separated to obtain platelet-poor plasma. All samples were quickly frozen in liquid nitrogen, stored at − 80 °C at the POLI-MI Biobank, Biological Resource Center of the Fondazione Ca’ Granda, and thawed at 37 °C for 3–4 min before being analyzed.

Indirect markers of the global coagulation activation such as fibrin degradation products together with markers of the fibrinolytic pathway and markers of activation of the extrinsic and the contact pathway were evaluated.

d-Dimer (DD), fibrin monomer complex (FM), and fibrin degradation products (FDP) levels were measured by the relevant commercial kits: Lias Auto d-Dimer (limit of detection 0.2 µg/mL, total imprecision < 4%), LIA FM (limit of detection 0.2 µg/mL, total imprecision < 5%), and Lias Auto P-FDP (limit of detection 2.5 µg/mL, total imprecision < 7.5%) (Sysmex Corporation, Kobe, Japan) on CS-2400 instrument (Sysmex Corporation) [22].

Plasma levels of FVII, FXI, and FXII were measured by one-stage assay using Synthasil on ACL Top instrument (Werfen, Bedford, MA, USA).

Activated FVII (FVIIa) levels were measured using the HemoclotTM Factor VIIa kit (Hyphen Biomed, France), a clotting method for the quantitative determination of FVIIa activity; the method is insensitive to FVII because the reagent used is the recombinant truncated human tissue factor (rTTF) unable to promote FVII activation [23]. The limit of detection of HemoclotTM Factor VIIa is < 1 mIU/mL and the total imprecision is CV < 7.3%.

Activated FXI (FXIa) was determined by the Biophen FXIa chromogenic assay (Hyphen Biomed, France); the commercial kit contains human FVIII, FIX, thrombin and FX, calcium, and synthetic phospholipids. All the reagents are present in excess, and the generated FXa is directly related to FXIa present in the tested sample and measured at 405 nm by its specific activity on an FXa chromogenic substrate (SXa-11) [24]. The detection limit of the Biophen FXIa assay is < 2.5 mIU/mL and the total imprecision CV < 8%.

Both FVIIa and FXIa assays were performed on CS-2400 instrument.

Activated FXII (FXIIa) concentrations were quantified by the CoaChrom FXII Chromogenic (CoaChrom Diagnostica GmbH, Austria) manual method: standard and test plasmas are diluted with the assay buffer containing a kallikrein inhibitor and the active protease FXIIa present in the samples cleaves a chromogenic substrate and releases *p*-nitroaniline (pNA), which can be measured photometrically at 405 nm. The results are calculated based on a curve obtained with a standard at known potency [25]. The standard curve is linear up to 1.5 U/mL. The total CV is < 10% and the detection limit 0.05 U/mL.

C1 esterase inhibitor (C1-INH) activity was quantified by a commercial chromogenic kit (Technochrom C1-INH, Technoclone GmbH, Vienna, Austria), with a detection limit of 6.6% and total imprecision CV < 5%.

Anti-factor Xa activity was evaluated using the Liquid Anti-Xa automated chromogenic assay (Werfen, Orangeburg, NY) on ACL Top, with a detection limit of 0.04 IU/mL and total imprecision CV < 4.0%.

Tissue-type plasminogen activator (t-PA) total antigen was measured in plasma using a commercially available ELISA (Zymutest t-PA antigen, Hyphen BioMed, Neuville sur Oise, France). Plasminogen activator inhibitor-1 (PAI-1) total antigen was measured in plasma using a commercially available ELISA (Zymutest PAI-1 antigen, Hyphen BioMed). For both the kits, intra- and inter-assay CVs is < 10%, and the lower detection limit is 0.5 ng/mL. Plasminogen and α2-antiplasmin (α2AP) plasma levels were measured on ACL Top instrument by the specific commercial chromogenic kits: HemosIL plasminogen (detection limit 2%; total imprecision CV < 2.7%) and HemosIL Plasmin Inhibitor (detection limit 7%; total imprecision CV < 5.9%), respectively.

All determinations were done in triplicates.

For FVII, FXI, FXII, plasminogen, a2AP, PAI-1, t-PA, and C1-INH, we used a normal reference range provided by commercial kits to define parameters alterations. For FVIIa, FXIa, FXIIa, DD, FM, and FDP, where normal range were not available, we used 100 healthy controls (50 males, 50 females) selected from a historical cohort of volunteers, all characterized by no personal history of thrombosis or thrombophilia abnormalities, arrived at our center in the pre-COVID-19 era to determine reference values.

### Statistical analysis

Clinical and routine laboratory data were obtained from electronic medical records. Mean and standard deviation (SD) or median and interquartile range (IQR) were used for continuous variables, and count and percentages were used to describe demographic and categorical clinical variables.

Comparisons of the coagulation parameters among patients at low, intermediate, and high intensity of care were performed using the Kruskal–Wallis non-parametric test.

For FVIIa, FXIa, and FXIIa, we performed a comparison between median values of COVID-19 patients and median values of the reference range with a non-parametric test. Moreover, the percentage of COVID-19 patients with plasma levels is higher than the median value of the reference range, alongside with their 95% Confidence Interval (CI) that has been calculated for each intensity of care group. This has been done for FVIIa, FXIa, and FXIIa, considering that the primary aim of the study was to assess the hypothesis of a predominant activation of the contact pathway of the coagulation cascade over the extrinsic pathway.

To evaluate the linear correlation between two sets of data the Spearman’s correlation coefficient (rho) was calculated. Stratification analysis by intensity of care, age, and occurrence of thrombotic events during hospital stay were also performed.

The level of statistical significance was set to 0.05 for all the performed tests.

## Results

A total of 111 COVID-19 patients (63% male) were consecutively included, 26 (23%) in low-intensity care wards, 42 (38%) in intermediate sub-intensive care wards and 43 (39%) in high-intensity care units. The mean age at enrollment was 59 (SD 12) with 34 (31%) patients older than 65 years (Table 1). Twelve patients (11%) died during the hospitalization.

**Table 1:** Demographic and clinical characteristics of the study population

Parameters	Patients tot (*n* = 111)	Group low (*n* = 26)	Group intermediate (*n* = 42)	Group high (*n* = 43)
Males, *n* (%)	70 (63)	13 (50)	28 (67)	29 (67)
Age, mean (SD)	59 (12)	60 (12)	59 (12)	60 (12)
Age < 65, *n* (%)	77 (69)	17 (65)	25 (60)	33 (77)
Age ≥ 65, *n* (%)	34 (31)	9 (35)	17 (40)	10 (23)
Thrombotic events, *n* (%)	32 (29)	5 (19)	15 (36)	12 (28)
Deaths, *n* (%)	12 (11)	0	6 (14)	6 (14)

*n* number, *SD* standard deviation

**Fig. 1:**
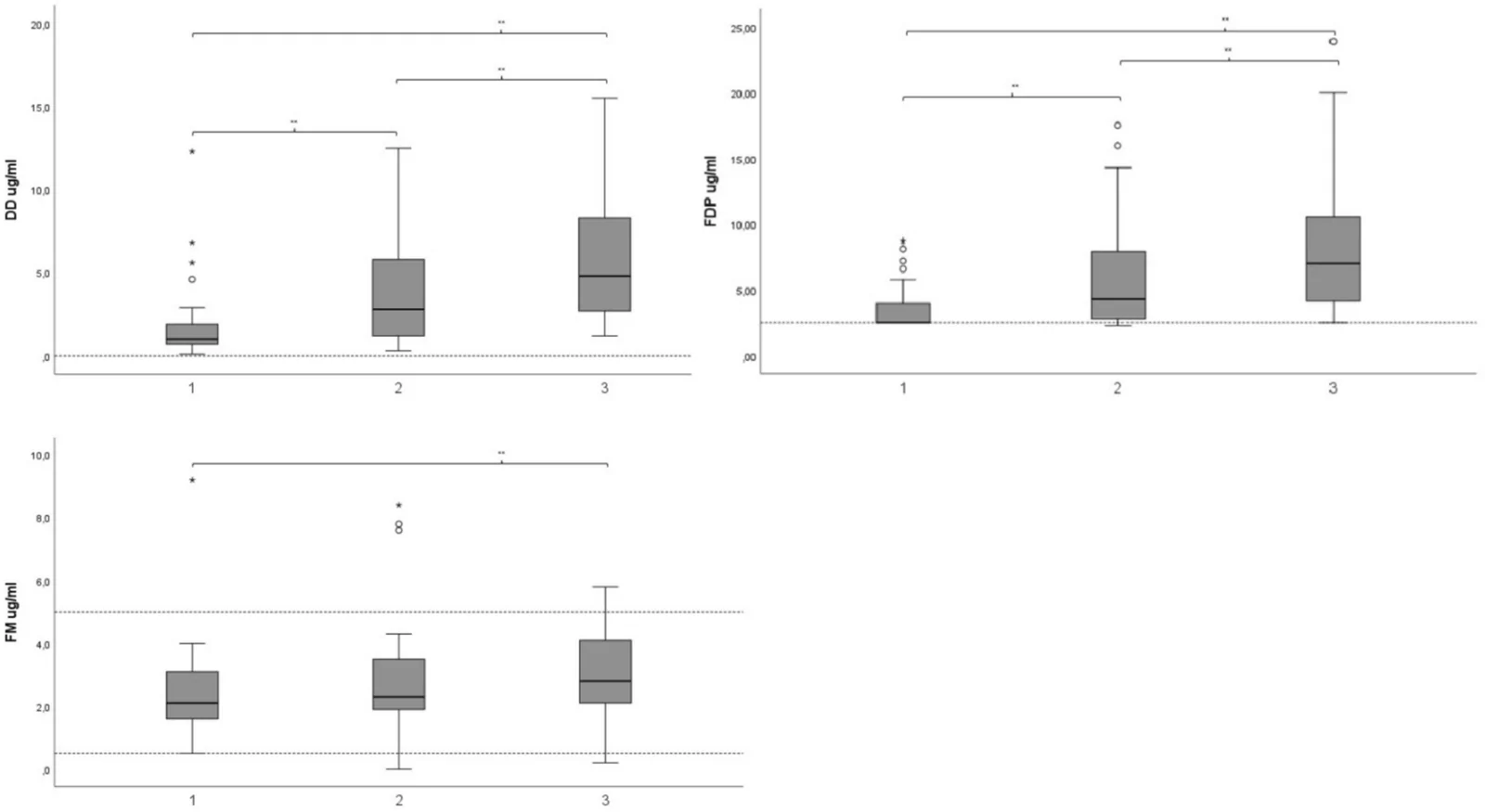
Box plots of the fibrin degradation products values in COVID-19 patients in the three intensity of care levels. On the *x*-axis are reported the patients’ categories (1, low-intensity of care COVID-19 patients; 2, intermediate-intensity of care COVID-19 patients; 3, high-intensity of care COVID-19 patients), while on the *y*-axis plasma levels of the tested parameters are reported (all in μg/mL). Dotted lines represent the reference range for each variable. **p < 0.005. *DD* d-dimer, *FDP* fibrin degradation product, *FM* fibrin monomer complex

Fibrin degradation products showed a gradient of increase from low to high-intensity care units. In particular, DD and FDP plasma levels were higher than the relevant reference cut-off in all patients (Table 2, Fig. 1).

**Table 2:** Distribution of the fibrin degradation products and of the parameters of the fibrinolytic system

Parameters	Reference range	Patients tot (*n* = 111)	Group low (*n* = 26)	Group intermediate (*n* = 42)	Group high (*n* = 43)
DD (µg/mL), median (IQR)	< 0.6	2.8 [1.2–5.6]	0.9 [0.7–1.4]	2.65 [1.2–5]	4.8 [2.7–8.4]
FDP (µg/mL), median (IQR)	< 2.5	4.5 [2.7–7.9]	2.4 [2.4–4.0]	3.9 [2.7–6.7]	7.0 [4.08–10.8]
FM (µg/mL), median (IQR)	0.5–5.2	2.3 [1.9–3.3]	2.0 [1.5–3.0]	2.2 [1.9–3.0]	2.6 [2.1–3.9]
PAI-1 Ag (ng/mL), mean (SD)	1.2–47	30.6 ± 10.2	29.7 ± 11.1	31.6 ± 10.0	30.3 ± 10.0
t-PA Ag (ng/mL), median (IQR)	2.9–18	16.1 [3.3–30.6]	17.4 [8.9–27.9]	30 [5.8–43.1]	5.8 [1.5–24.3]
α2AP (%), mean (SD)	98–122	117.4 ± 13.2	115.6 ± 14.4	116.6 ± 11.2	119.3 ± 14.3
PLG (%), mean (SD)	80–132	109.6 ± 20.0	109.6 ± 24.4	109.9 ± 16.5	114.4 ± 20.6

*N* number, *DD* d-dimer, *FDP* fibrin degradation product, *FM* fibrin monomer complex, *PAI-1* plasminogen activator inhibitor-1, *Ag* antigen, *t-PA* tissue-type plasminogen activator, *α2AP* alpha2-antiplasmin, *PLG* plasminogen, *IQR* interquartile range, *SD* standard deviation

**Fig. 2:**
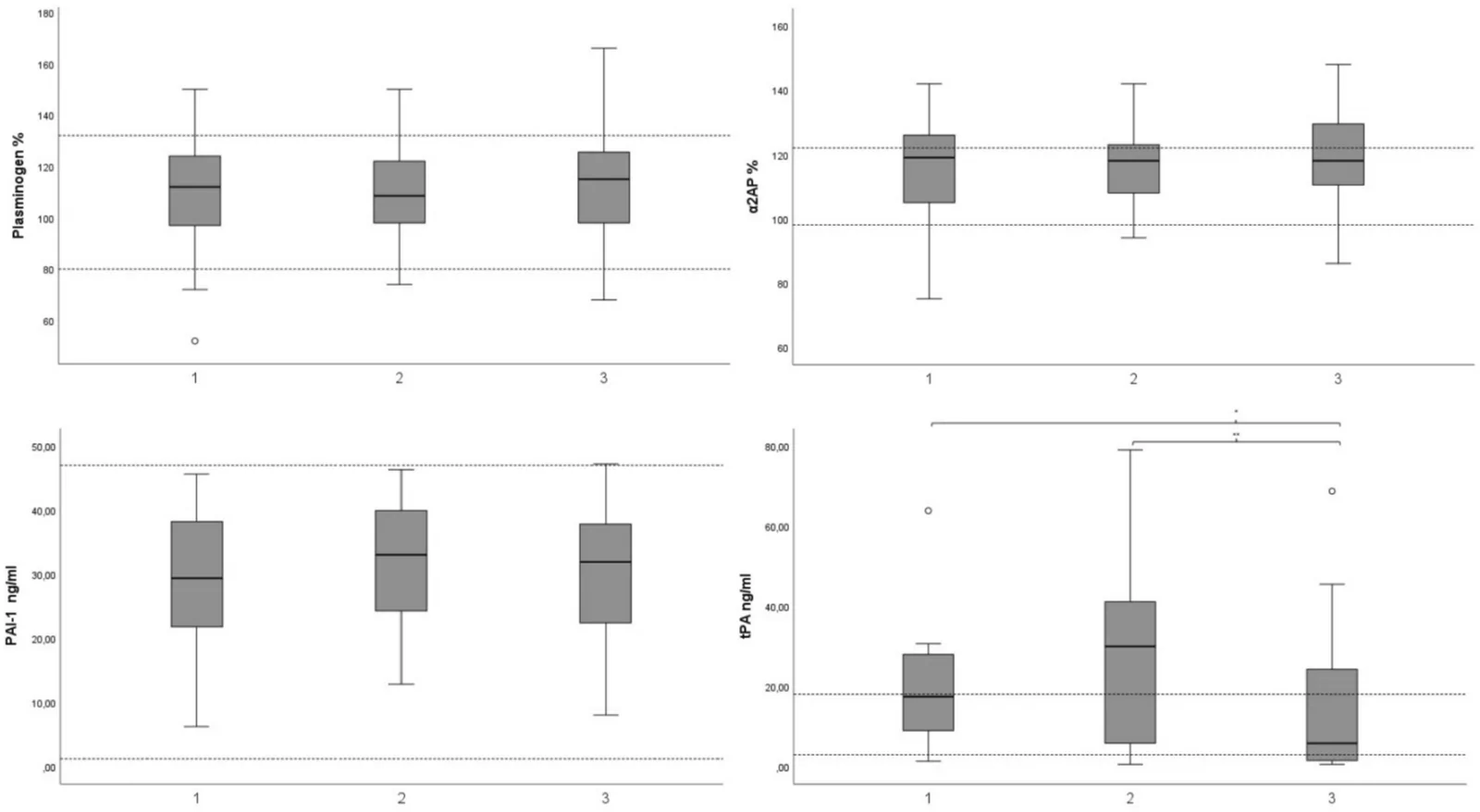
Box plots of the parameters of the fibrinolytic system. On the *x*-axis are reported the patients’ categories (1, low-intensity of care COVID-19 patients; 2, intermediate-intensity of care COVID-19 patients; 3, high-intensity of care COVID-19 patients), while on the *y*-axis plasma levels of the tested parameters are reported. Dotted lines represent the reference range for each variable. *p < 0.05, **p < 0.005. *PAI-1* plasminogen activator inhibitor-1, *t-PA* tissue-type plasminogen activator, *α2AP* alpha2-antiplasmin, *PLG* plasminogen

All the fibrinolytic pathway parameters were in the normal range, with no significant differences between the three intensity groups, except t-PA for which we found increased levels in the low- and intermediate-intensity groups compared to the high-intensity group (Table 2, Fig. 2).

Plasma levels of non-activated FVII (reference interval 62–138%), FXI (reference interval 70–130%) and FXII (reference interval 70–130%) were within the normal range in all patients.

**Fig. 3:**
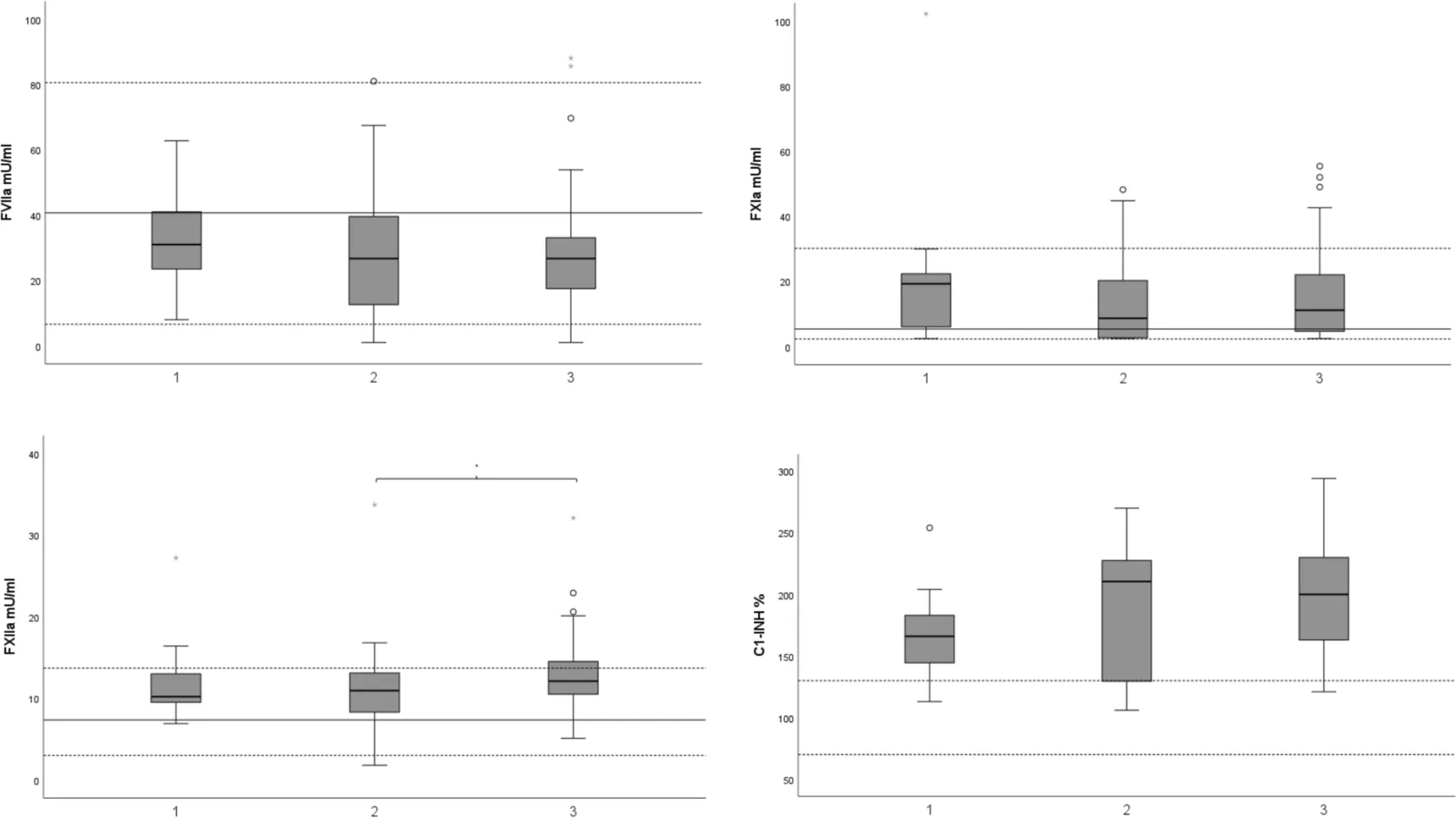
Box plots of the markers of extrinsic (FXIIa) and contact pathway (FXIa, FXIIa, C1-INH) of the coagulation cascade in COVID-19 patients in the three intensity of care levels. On the *x*-axis are reported the patients’ categories (1, low-intensity of care COVID-19 patients; 2, intermediate-intensity of care COVID-19 patients; 3, high-intensity of care COVID-19 patients), while on the *y*-axis plasma levels of the tested parameters are reported. Dotted lines represent the reference ranges. For FVIIa, FXIa and FXIIa median values of the reference ranges are reported as solid lines. Dotted lines represent the reference range for each variable. *p < 0.05. *FVIIa* activated factor VII, *FXIa* activated factor XI, *FXIIa* activated factor XII, *C1-INH* C1 inhibitor

Overall, median values of FVIIa, FXIa, and FXIIa in COVID-19 patients were significantly different from those extrapolated from the reference range (*p* < 0.01). Although FVIIa values were within the normal range in COVID-19 patients, we observed 19.8% (95% CI 13.2–28.0) of patients with FVIIa plasma levels higher than the median value of the reference range (40.7 mU/mL). No statistically significant differences were observed between the three intensity care units (Fig. 3).

Plasma levels of FXIa and FXIIa were within the reference intervals in the majority of the patients, with 68.5% (95% CI 59.4–76.6) and 91% (95% CI 84.5–95.3) of patients over the median FXIa and FXIIa reference values, respectively (Table 3, Fig. 3). No statistically significant differences were observed between the three intensity care groups for FXIa, while FXIIa showed a statistically significant slight gradient of increase between the intermediate- and high-intensity care units (from 10.9 to 11.9 mU/mL).

**Table 3:** Distribution of the markers of extrinsic (FXIIa) and contact pathway (FXIa, FXIIa, C1-INH) of the coagulation cascade

Parameters	Reference range	Patients tot (*n* = 111)	Group low (*n* = 26)	Group intermediate (*n* = 42)	Group high (*n* = 43)
FVIIa (mU/mL), median (IQR)	6.6–80.6	27.5 [16.5–38.9]	29.9 [22.9–40.7]	26.7 [12.6–39.6]	26.7 [17.5–33.5]
FXIa (mU/mL), median (IQR)	2.4–30.2	11.3 [4–22.3]	19 [4.9–22.1]	8.7 [2.7–20.3]	11.2 [4.7–22.4]
FXIIa (mU/mL), median (IQR)	3.0–13.7	11.15 [9.4–13.25]	10 [9.1–12.5]	10.9 [8.3–12.9]	11.9 [10.2–14.2]
C1-INH (%), mean (SD)	70–130	180.5 ± 47.4	164.1 ± 34.2	197.0 ± 49.1	198.4 ± 43.0

*FVIIa* activated factor VII, *FXIa* activated factor XI, *FXIIa* activated factor XII, *C1-INH* C1 inhibitor, *IQR* interquartile range, *SD* standard deviation

C1-INH plasma levels were increased in COVID-19 patients and significantly higher in the intermediate- and high- compared to the low-intensity care group (Table 3, Fig. 3).

A restriction analysis for the presence of thrombosis for each considered parameter showed no statistically significant influence (Supplementary Figure).

Similarly, when we restricted the analysis to patients younger than 65 years, excluding the thirty-nine older patients, no statistically significant differences were found suggesting that advanced age was not responsible for the main findings.

As expected, the three fibrin degradation products (DD, FDP, FM) showed a positive linear correlation (rho > 0.6, *p* < 0.0001), while no linear correlation was found between FXIIa or FXIa and fibrin degradation products. The same analysis was conducted with other methods as well (i.e., logarithmic, inverse, quadratic and cubic), showing no significant correlation (*R*^2^ < 0.007). FXIIa plasma levels were found to correlate positively with plasminogen (rho 0.236, *p* 0.016), PAI-1 (rho 0.232, *p* = 0.02), and α2AP (rho 0.197, *p* = 0.04), showing a direct relationship between contact pathway activation and fibrinolytic cascade. As to fibrin degradation products and fibrinolysis parameters, t-PA is the only fibrinolytic parameter that showed a negative correlation with DD (rho − 0.342, *p* = 0.0007), FDP (rho − 0.397, *p* = 0.0001), and FM (rho − 0.233, *p* = 0.019). C1-INH positively correlated with DD (rho 0.470, *p* < 0.0001) and FDP (rho 0.394, *p* = 0.0001).

## Discussion

The main pathophysiologic mechanism leading to the unrestricted activation of coagulation during SARS-CoV-2 infection has not been elucidated yet. Given the important inflammatory response induced by the virus [26] and the close interplay between inflammation and hemostasis [12] and based on the results of previous studies showing an activation of the contact pathway [21, 27–29], we hypothesized a prominent activation of this pathway over the extrinsic pathway in COVID-19 patients. Indeed, the contact pathway of the coagulation cascade may be activated directly by the SARS-CoV-2 virus [30] or indirectly by the induced inflammation response.

By evaluating FVIIa as a marker of activation of the extrinsic pathway of the coagulation cascade, we found its plasma levels to decrease in COVID-19 patients with only 19.8% (95% CI 13.2–28.0) of patients with higher plasma levels than the median value of the reference values. This observation could be a consequence of a reduced production due to a relatively lower activation of the extrinsic pathway or to a consumption of FVIIa due to the binding with tissue factors or other molecules on platelets or leucocyte plasma membranes. Indeed, tissue factors have been reported very early on to be elevated in COVID-19 patients [31–33]. We speculate that in an initial phase of the disease, activation of the extrinsic pathway as a consequence of the endothelial damage directly induced by the virus itself and indirectly by the inflammatory response represents the main activation pathway of the coagulation cascade [18]. In the second phase, in the condition of sustained inflammatory response, the contact pathway is activated as well and becomes the main mechanism responsible for the coagulation activation [34]. This hypothesis is consistent with the findings of the present study, where patients with overt SARS-CoV-2 infection have been included, and the blood samples were obtained many days after the symptoms’ onset. Pre-analytical factors could have also contributed to explain decreased FVIIa levels. Indeed, FVIIa has a very short half-life; therefore, the time from blood sampling and centrifugation is crucial [35]. However, all the collected blood samples have been centrifuged within 2 h, abolishing the confounding effect of this pre-analytical error. Therefore, the reduction in FVIIa plasma levels in COVID-19 patients was likely accounted for by the underlying pathophysiological mechanism, leading to a diminished production or half-life in vivo.

On the other hand, by evaluating FXIIa and FXIa as markers of the activation of the contact pathway of the coagulation cascade, we found increased plasma levels in COVID-19 patients, with 68.5% (95% CI 59.4–76.6) and 91% (95% CI 84.5–95.3) of patients with higher FXIa and FXIIa plasma levels than the median value of the reference values, respectively. These findings suggest that FXII-mediated activation of the contact pathway is increased in patients with severe COVID-19 requiring hospitalization, potentially contributing to the prothrombotic and inflammatory complications of SARS-CoV-2 infection, as demonstrated in other conditions characterized by an extensive thromboinflammation such as sickle cell disease or pancreatic cancer [36, 37]. Indeed, there is growing evidence of the crucial role of FXIIa-mediated thromboinflammation in various pathological states other than classical thrombosis, such as vascular inflammation [38]. It must be said that the methods used for the determination of FXIIa and FXIa plasma levels are not reliable when the plasma levels of heparin are higher than 0.5 and 0.2 U/mL in terms of anti-Xa activity, respectively. This interference is due to the physiological in vivo mechanism of action of heparin, that inhibits, other than FXa and FIIa, also FIXa and FXIa, even though to a minor extent. Due to the physiologic negative feedback of FXIa on FXIIa, the inhibition of FXIa by heparin results also in an indirect inhibition of FXIIa. Moreover, the method for FXIa plasma level determination is affected by the presence of heparin in the sample due to the method itself, which is a chromogenic assay evaluating the residual anti-Xa activity. To establish the reliability of the FXIa and FXIIa results, considering that heparin may affect these test results, factor Xa activity has been evaluated on each blood sample; however, considering that anti-Xa activity was not an investigational biological marker in our study, we decided not to systematically report these data. Anti-Xa activity due to heparin in patients’ plasma was between 0 and 1.38 U/mL, with only 10 patients showing an anti-Xa activity > 0.5 and only 6 patients > 1.0 U/mL. Therefore, the overall impact of heparin on FXIIa determination was negligible. On the other hand, a significant proportion (30 patients) of plasma samples had an anti-Xa activity > 0.2 U/mL, making the test for FXIa unreliable on the actual values of this parameter in the tested samples. More in detail, the presence of heparin reduces the detected concentration of FXIa, but in a non-linear way, avoiding the possibility to identify a correction factor to determine the actual levels of FXIa. For this reason, this method underestimates the plasma levels of FXIa in the presence of heparin. Therefore, the finding of increased plasma levels of FXIa in COVID-19 patients is reliable and represents an underestimation of the true in vivo concentration. However, a comparison of FXIa levels between the three intensity care groups is not feasible since all patients were on treatment with heparin and, as stated above, no linear correlation between heparin levels in terms of anti-Xa activity and FXIa levels does exist. Other than heparin, there are other FXIa inhibitors in plasma, including antithrombin, glycosaminoglycans, C1-INH, protease nexin-1, and protein Z-dependent protease inhibitor/protein Z complex [39]. No data on the impact of these inhibitors during the assay performance, and during the sample handling are available.

The plasma levels of the non-activated forms of FVII, FXI, and FXII were determined in all enrolled patients to exclude congenital or acquired deficiencies of these clotting factors that would have an impact on the results of the relative active form.

C1-INH is a serine protease that represents the major inhibitor of the contact pathway and its deficiency has been associated to increased levels of circulating markers of coagulation activation and increased risk of venous thrombosis in both mice models and humans [40]. The increased levels of C1-INH found in patients with overt COVID-19 disease are coherent with its prominent role as a negative regulator of the contact pathway of the coagulation cascade. On the other hand, it may have contributed to mitigate the observed level of activation of the contact pathway in our cohort of patients.

Given the observation of low plasma levels of t-PA in patients admitted to low-intensity care, which become high in intermediate and again low in high-intensity care, we may argue that the three intensity care groups reflect different times of activation in the hemostatic steps. In the first phases of the disease, the coagulation pathway is starting to activate; in the second phase, the fibrinolytic pathway activates to an extent related to the grade of coagulation activation and then both processes slow down.

The time of in-hospital diagnosis was not recorded though it does not differ significantly from the time of admission, being the period in the emergency department limited to up to 3 days (median 1 day, range 0–3 days). Time since admission was recorded for all patients and ranged from 0 to 31 days (median 8 days).

The influence of acute thrombosis on coagulation parameters is well known. In our cohort, 32 patients out of 111 (29%) had a thrombotic event during hospitalization, potentially influencing the investigated biological parameters. All of them developed a venous thromboembolic event and one of these patients also had an ischemic stroke. Moreover, among patients who developed thrombosis, 14 (44%) were sampled after the thrombotic event. Among these 14 patients, blood sampling took place with a median of 6.5 days after the thrombotic event (range 1–19 days). The low absolute number of thrombotic events did not allow us to perform an association analysis in this subgroup of patients, also considering that this was not the aim of the study. However, we restricted the analysis for the presence of thrombosis for each considered parameter, showing results similar to the main findings (Supplementary Figure). No stratification for intensity of care level has been performed due to the low number of patients in each group.

Together with thrombosis, many other medical conditions can influence the coagulation parameters, such as cardiovascular or immunologic diseases. Data on comorbidities were not systematically collected in our study so that adjustment for these data has not been possible. However, considering age as the main risk factor for thrombosis and for the presence of comorbidities, we also restricted the analysis to patients younger than 65 years, without observing any difference in the results. Due to the limited number of patients, a sub-analysis for the three care-intensity groups was not performed.

The lack of correlation between the FXIIa or FXIa and the fibrin degradation products does not rule out a lack of association between the extrinsic and the contact pathway on the one hand, and the global coagulation activation on the other hand, but simply that this relationship may not be well described through correlation. Indeed, there is no direct association between these two mechanisms, but many intermediate passages (not evaluated) do exist. Moreover, these are not simultaneous mechanisms, but one is the result of the activation of the first two, while they have all been tested on the same plasma sample reflecting only one time point.

FXIIa plasma levels were found to correlate positively with plasminogen, PAI-1 and α2AP, showing a direct relationship between contact pathway activation and the fibrinolytic cascade, both the activator (plasminogen) and the inhibitory patterns (PAI-1 and α2AP). Indeed, the close relationship between the contact pathway and the fibrinolytic pathway has been described [41, 42]. The main link is represented by kallikrein that directly converts plasminogen to plasmin, the effector protein of the fibrinolytic pathway, but also FXIa and FXIIa, even though at a minor extent, are able to activate plasminogen to plasmin. Moreover, thrombin generation resulting from the activation of the contact pathway is also responsible for the dampening of the fibrinolytic system via activation of the thrombin-activatable fibrinolysis inhibitor (TAFI). Moreover, there is also a negative feedback of the fibrinolytic system on the contact pathway carried out by PAI-1 on FXIIa [34].

The absence of a correlation between fibrin degradation products and fibrinolysis parameters corroborates the hypothesis that the increased levels of fibrin degradation products are a consequence of the coagulation cascade activation and not secondary to hyper-fibrinolysis. t-PA is the only fibrinolytic parameter that showed a negative correlation with DD, FDP, and FM, supporting the above reported hypothesis. C1-INH positively correlated with DD and FDP, in line with the central role of C1-INH in negatively regulating the activated contact pathway. However, it should be stated that, other than the correlation between the three fibrin degradation products that appeared to be strong (rho > 0.6), all the other reported positive or negative correlations were weak to moderate (rho range 0.2–0.6).

Considering that the three different groups of patients at different intensity care potentially reflect different steps of activation of the hemostatic response, a correlation analysis between the biological parameters of the different hemostatic pathways should be preferentially performed within each group. However, the limited sample size for each group did not allow us to perform such an analysis with a statistical validity.

We would like to highlight the fact that, despite the official ISTH guidelines for anti-thrombotic prophylaxis in COVID-19 patients came out in July 2020, based on the observation of unexpectedly high rates of venous thromboembolic events in this population, a well-established protocol for anti-thrombotic prophylaxis was conceived early on during the COVID-19 pandemic at our center, making more homogeneous our dataset. This was previously reported by our group [43].

We recognize that this study has some limitations that we would like to highlight. Due to the emergency setting that characterized the first wave of the COVID-19 pandemic, detailed clinical features of the patient cohort were not prospectively collected; so that, only very limited clinical data, such as sex, age and oxygen need, are available. The absence of a control group represents a limit of this study; however, this study was not designed as a case–control but as a cross-sectional study with the aim to evaluate the differential activation of the extrinsic and the contact pathway in COVID-19 patients other than comparing this activation with a control population. As a consequence of this design, it is not possible to evaluate the potential role of factors other than SARS-CoV-2 infection itself, such as medical respiratory devices that were used in some patients, on the activation of the contact pathway. We should recognize that our study is not all-embracing: many other activation biomarkers, such as tissue factor, and markers of complement activation and thrombo-inflammation, have not been tested and the protease/inhibitor complexes, globally indicative of the contact pathway activation, have not been evaluated. However, at least one biomarker of activation for each pathway of the coagulation cascade has been analyzed, together with the main inhibitor of the contact pathway (C1-INH). Despite the unavoidable lack of completeness, our study contributes, together with other available data, to elucidate the modalities of activation of the coagulation cascade in COVID-19 and, potentially, in other similar settings. Global coagulation assays such as thrombin generation have not been tested due to interference of heparin contained in the blood samples. Therefore, the activation of the coagulation cascade has been indirectly evaluated by testing the fibrin degradation products levels together with markers of the fibrinolytic pathway activation.

## Conclusions

Overall, the results of our study suggest a predominant activation of the contact pathway over the extrinsic pathway of the coagulation cascade in patients with severe COVID-19 requiring hospitalization. Considering the reported concomitant high risk of bleeding in these patients while on anticoagulant treatment, together with the high risk of thrombosis, the evidence of a crucial role of the contact pathway in coagulation activation is of remarkable importance. Indeed, drugs targeting this pathway, that are efficacious in terms of thrombosis recanalization without an increase of the rate of bleeding events, are currently under advanced phases of investigation and may be promising in this form of coagulopathy to limit the bleeding risk while treating thrombosis and concurrently reducing inflammation and the associated complications.

Despite 5 years have passed form the pandemic, we believe COVID-19 has represented a valid study model for better understanding the contact pathway with the aim to better identify in future the subset of patients that may benefit from inhibitors of this pathway.

## Supplementary Information

Below is the link to the electronic supplementary material.

Supplementary file1 (DOCX 156 KB)

## Data Availability

The data that support the findings of this study are available from the corresponding author upon reasonable request.
