# Supplementary material for: Involvement of the contact pathway in COVID-19 coagulopathy
Source: Intern Emerg Med. 2025 Nov 8;21(7):2699–709. doi: 10.1007/s11739-025-04191-z (PMC13694401; doi:10.1007/s11739-025-04191-z)
Supplement: Supplementary file 1 [file 11739_2025_4191_MOESM1_ESM.docx]

**Supplementary Figure. Box plots showing no influence of thrombosis on fibrin degradation products, FXIa, FXIIa and FVIIa.** On the x axis are reported (from left to right) the reference group (Ref), COVID-19 patients without thrombosis (1), COVID-19 patients who developed thrombosis with the plasma sample collected before thrombosis onset (2) and COVID-19 patients who developed thrombosis with the plasma sample collected after thrombosis onset (3), while on the y axis plasma levels of the tested parameters are reported (ug/mL for DD, FDP and FM, and mU/mL for FXIa, FXIIa and FVIIa).

FVIIa, activated factor VII; FXIa, activated factor XI; FXIIa, activated factor XII; DD, D-dimer; FDP, fibrin degradation product; FM, fibrin monomer complex.
